# Near-Infrared Photoimmunotherapy Combined with CTLA4 Checkpoint Blockade in Syngeneic Mouse Cancer Models

**DOI:** 10.3390/vaccines8030528

**Published:** 2020-09-14

**Authors:** Yasuhiro Maruoka, Aki Furusawa, Ryuhei Okada, Fuyuki Inagaki, Daiki Fujimura, Hiroaki Wakiyama, Takuya Kato, Tadanobu Nagaya, Peter L. Choyke, Hisataka Kobayashi

**Affiliations:** Molecular Imaging Program, Center for Cancer Research, National Cancer Institute, NIH, Bethesda, MD 20892, USA; ymaruoka@med.kyushu-u.ac.jp (Y.M.); Aki.Furusawa@nih.gov (A.F.); Ryuhei.Okada@nih.gov (R.O.); Fuyuki.Inagaki@nih.gov (F.I.); d-fuji@shimadzu.co.jp (D.F.); Hiroaki.Wakiyama@nih.gov (H.W.); Takuya.Kato@nih.gov (T.K.); nagaya@shinshu-u.ac.jp (T.N.); pchoyke@mail.nih.gov (P.L.C.)

**Keywords:** near infrared photoimmunotherapy, immune checkpoint blockade, CTLA4, CD44, monoclonal antibodies

## Abstract

Near infrared photoimmunotherapy (NIR-PIT) is a newly developed and highly selective cancer treatment that induces necrotic/immunogenic cell death. It employs a monoclonal antibody (mAb) conjugated to a photo-absorber dye, IRDye700DX, which is activated by NIR light. Tumor-targeting NIR-PIT is also at least partly mediated by a profound immune response against the tumor. Cytotoxic T-lymphocyte antigen-4 (CTLA4) is widely recognized as a major immune checkpoint protein, which inhibits the immune response against tumors and is therefore, a target for systemic blockade. We investigated the effect of combining tumor-targeted NIR-PIT against the cell-surface antigen, CD44, which is known as a cancer stem cell marker, with a systemic CTLA4 immune checkpoint inhibitor in three syngeneic tumor models (MC38-luc, LL/2, and MOC1). CD44-targeted NIR-PIT combined with CTLA4 blockade showed greater tumor growth inhibition with longer survival compared with CTLA4 blockade alone in all tumor models. NIR-PIT and CTLA4 blockade produced more complete remission in MOC1 tumors (44%) than NIR-PIT and programmed cell death protein 1 (PD-1) blockade (8%), which was reported in our previous paper. However, the combination of NIR-PIT and CTLA4 blockade was less effective in MC38-luc tumors (11%) than the combination of NIR-PIT and PD-1 blockade (70%). Nonetheless, in many cases ineffective results with NIR-PIT and PD-1 blockade were reversed with NIR-PIT and CTLA4 blockade.

## 1. Introduction

Near infrared photoimmunotherapy (NIR-PIT) is a newly developed cancer treatment that induces highly specific cell death in targeted tumor cells. It employs a monoclonal antibody (mAb) conjugated to a silica-phthalocyanine photoabsorbing dye, IRDye700DX (IR700) [1,2]. This antibody-IR700 conjugate is administered intravenously, and after a suitable time to allow for target binding, the tumor is exposed to 690 nm NIR light which activates IR700 [3]. NIR-PIT induces stress on the cellular membrane through photoinduced ligand-release of IR700, leading to impaired membrane function, rapid volume expansion with cell membrane rupture and extrusion of the cell contents into the extracellular space [4,5]. NIR-PIT has an important advantage over other cancer therapies, and induces highly selective necrotic/immunogenic cell death to tumor cells with minimal damage to adjacent normal cells unlike most other conventional cancer therapies that induce apoptotic cell death in both cancer and normal cells [6,7]. A phase III clinical trial of NIR-PIT using the epidermal growth factor receptor (EGFR) -targeted antibody-IR700 conjugate, cetuximab-IR700, in patients with inoperable head and neck cancer is underway (https://clinicaltrials.gov/ct2/show/NCT03769506).

CD44 is a well-known marker of cancer stem cells [8] and anti-CD44-mAb-IR700 NIR-PIT induces effective tumor killing in CD44-expressing syngeneic mouse models [9,10,11]. Recently, the combination of CD44-targeted NIR-PIT and programmed cell death protein 1 (PD-1) immune checkpoint blockade was reported to induce massive tumor cell death and innate priming of polyclonal, cancer antigen-specific T-cell responses in several cancer models, resulting in complete tumor rejection and the generation of immunologic memory [10]. However, in some cancer models, CD44-targeted NIR-PIT combined with PD-1 blockade did not show benefits over CD44-targeted NIR-PIT alone.

Cytotoxic T-lymphocyte antigen-4 (CTLA4) is a widely recognized immune checkpoint protein, which inhibits the immune response against tumors [12,13,14], down-regulating T cell activation in response to engagement of the T cell receptor. CTLA4 is expressed transiently on activated T cells, peaking at 2–3 days after initial activation [15], and is always strongly expressed on regulatory T cells (Tregs). Blockade of the interaction between CTLA4 and its ligands, CD80/86 using anti-CTLA4 mAbs can reduce Treg populations leading to increase in effector T cells, thus enhancing anti-tumor immunity [16,17]. CTLA4 and PD-1 blockade, although both intended to enhance anti-tumor immunity, produce distinct immunoregulatory patterns that vary in effectiveness with each tumor model. Therefore, we hypothesized that NIR-PIT combined with CTLA4 blockade could achieve different and perhaps complementary therapeutic effects from NIR-PIT combined with PD-1 blockade. The purpose of this study was to investigate the in vivo therapeutic efficacy of the combination of CD44-targeted NIR-PIT and CTLA4 blockade in several syngeneic mouse models of cancer.

## 2. Results

### 2.1. Comfirmation of CD44 Expression as a Target for NIR-PIT and In Vitro Tumor Cell Killing of CD44-Targeted NIR-PIT in MC38-luc, LL/2, and MOC1 Tumor Cells

**Figure 1:**
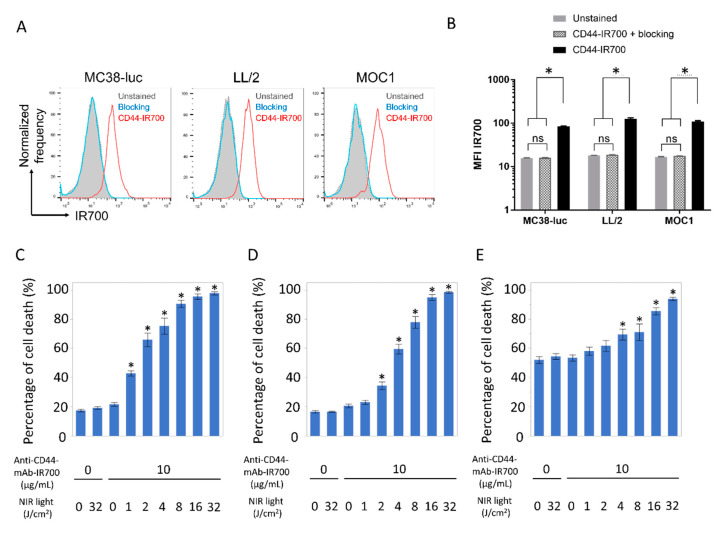
Confirmation of CD44 expression as a target for NIR-PIT and evaluation of in vitro CD44-targeted NIR-PIT in MC38-luc, LL/2, and MOC1 cells. (**A**) Expression of cell-surface CD44 in MC38-luc, LL/2 and MOC1 cells was examined with flow cytometry. CD44-blocking antibody was added to some wells to validate specific staining. Representative histograms were shown. (**B**) Mean fluorescence intensity (MFI) of IR700 after labeling with anti-CD44-mAb-IR700 (n = 4, * *p* < 0.05, Tukey–Kramer test). (**C**–**E**) Membrane permeability as measured by PI staining, after labeling with anti-CD44-mAb-IR700 and treatment with NIR light. (**C**) MC38-luc; (**D**) LL/2; (**E**) MOC1 (n = 5, * *p* < 0.05, vs. untreated control; Mann–Whitney U test). ns, not significant.

To confirm CD44 expression as a target for NIR-PIT and tumor cell killing effects by CD44-targeted NIR-PIT, flow cytometry and in vitro CD44-targeted NIR-PIT were performed in the three different types of tumor cells (Figure 1A–E). Flow-cytometric analysis revealed high fluorescent signal intensity in MC38-luc, LL/2, and MOC1 cells after exposure to anti-CD44-mAb-IR700 (Figure 1A,B). This signal was completely reversed in the presence of excess unconjugated anti-CD44-mAb, verifying binding specificity (Figure 1A,B). Based on incorporation of propidium iodide (PI), the percentage of cell death increased in a light dose dependent manner in MC38-luc, LL/2, and MOC1 cells (Figure 1C–E). There was no significant cytotoxicity associated with NIR light alone in the absence of anti-CD44-mAb-IR700 and with anti-CD44-mAb-IR700 alone without NIR light (Figure 1C–E). In MOC 1 cells, relatively high percentage of cells were dead even in untreated samples. This was probably because hash handling was needed to detach MOC1 cells from the plate with a cell scraper due to the strong adhesion.

### 2.2. Comparison of CD44 Expression among MC38-luc, LL/2, and MOC1 Tumors

**Figure 2:**
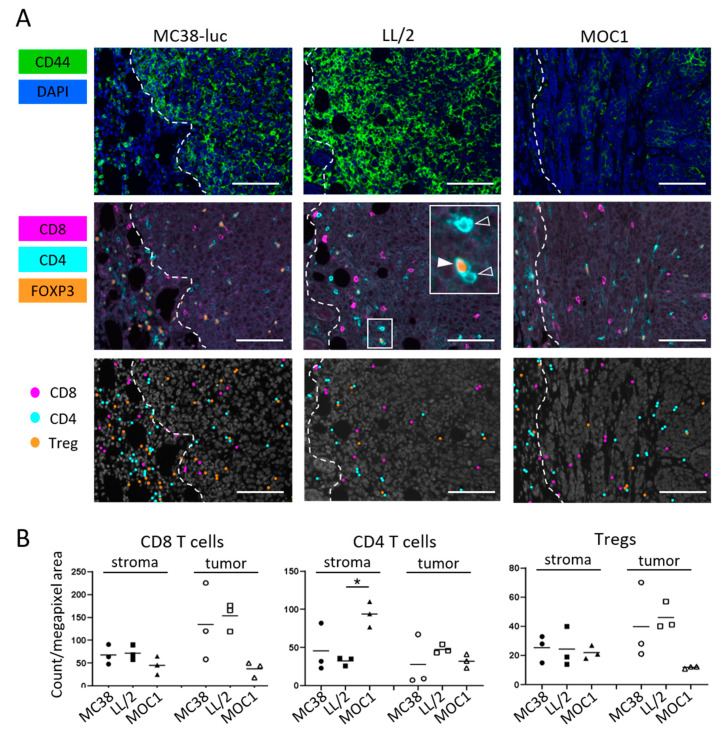
CD44 expression and immune cell infiltration within MC38-luc, LL/2, and MOC1 tumors. (**A**) Multiplex immunohistochemistry (IHC) staining was performed to examine CD44 expression and distribution of T cells within MC38-luc, LL/2, and MOC1 tumors before treatment. The top panels show CD44 expression (shown in green). Nucleus were stained with DAPI (shown in blue). White dotted lines represent tumor edges. The middle panels show expressions of CD8 (magenta), CD4 (cyan) and FOXP3 (orange). Inset shows examples of non-regulatory CD4 T cells (membrane CD4^+^/nucleus FOXP3^−^, open arrowhead) and Treg (membrane CD4^+^/nucleus FOXP3^+^, filled arrowhead). The bottom panels show cellular phenotypes based on the antigen expressions. CD8 T cell, non-regulatory CD4 T cells, Tregs are shown as dots in magenta, cyan and orange respectively. Representative images from at least three samples are shown (×200, scale bar = 100 µm). (**B**) T cell count in the tumor microenvironment. Cell number of CD8 T cells, non-regulatory CD4 T cells and Tregs in stroma and tumor tissue were counted in multiplex IHC images. Data were shown as cell count per megapixel area (n = 3; * *p* < 0.05; Tukey’s multiple comparison test).

To compare differences in CD44 expression in vivo among the three different types of tumors, size-matched tumors were assessed by means of immunohistochemical (IHC) staining (Figure 2A). CD44 expression was lower in MOC1 tumor tissues than MC38-luc and LL/2 tumors, which has also been demonstrated by flow cytometry analysis in the previous study [10]. The previous study also showed low fluorescence intensity of anti-CD44-mAb-IR700 in MOC1 tumors with quantitative analysis [10]. These suggest that tumor accumulation of anti-CD44-mAb-IR700 1 day after injection as a target antigen is predicted to be lower in MOC1 tumors and direct cell killing by CD44-targeted PIT is expected to be less efficient compared with MC38-luc or LL/2 tumors. We also examined distribution of T cells in the tumor microenvironment of three tumor types. Multiplex IHC showed all three types of tumor had infiltration of CD8 T cells, non-regulatory CD4 T cells and Tregs (Figure 2A). MOC1 tumor had significantly more non-regulatory CD4 T cells in the stroma, also, although statistically not significant, MOC1 had tendency to have smaller number of CD8 T cells and Tregs infiltrating into the tumor (Figure 2B).

### 2.3. Efficacy of CD44-Targeted NIR-PIT Combined with CTLA4 Blockade for MC38-luc Tumor

**Figure 3:**
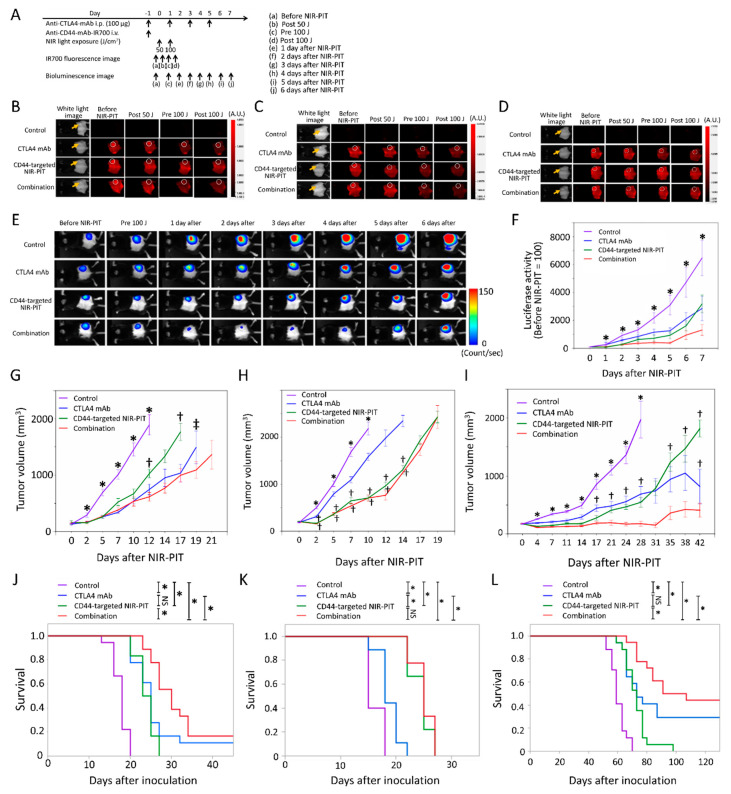
In vivo effects of CD44-targeted NIR-PIT and/or CTLA4 mAb administration for MC38-luc, LL/2, and MOC1 tumor models. (**A**) NIR-PIT regimen. Bioluminescence and fluorescence images were obtained at each time point as indicated. (**B**–**D**) Real-time in vivo IR700 fluorescence imaging of tumor-bearing mice before and approximately 10 min after NIR-PIT in MC38-luc (**B**), LL/2 (**C**) and MOC1 (**D**) tumor models. The yellow arrows and white circles indicate the tumor locations. (**E**) In vivo bioluminescence imaging of MC38-luc tumor-bearing mice before and after treatment at the indicated timepoints. (**F**) Quantitative analysis of luciferase activity before and after treatment in MC38-luc tumor-bearing mice. n ≥ 10/group, mean ± SEM; * *p* < 0.05, vs. the other groups; Dunnett’s test. (**G**–**I**) Tumor growth in all the groups for MC38-luc (**G**), LL/2 (**H**) and MOC1 (**I**) tumor models. n ≥ 9/group, mean ± SEM; * *p* < 0.05, vs. the other groups; Dunnett’s test; ^†^ *p* < 0.05, vs. combination group; Tukey–Kramer test; ^‡^ *p* < 0.05, vs. combination group; Mann–Whitney U test. (**J**–**L**) Survival curves in all the groups for MC38-luc (**J**), LL/2 (**K**) and MOC1 (**L**) tumor models. n ≥ 9/group; * *p* < 0.05; NS, not significant; Gehan–Breslow–Wilcoxon test.

The NIR-PIT regimen and imaging protocol are depicted in Figure 3A. In the NIR-PIT treated groups, anti-CD44-mAb-IR700 was administrated one day before the first light exposure (day −1), and the NIR-light was exposed over the following two days; 50 J/cm^2^ on day 0 and 100 J/cm^2^ on day 1 (24 and 48 h after anti-CD44-mAb-IR700 injection, respectively). IR700 fluorescence signal in tumors decreased due to dispersion of fluorophore from dying cells and partial photo-bleaching (Figure 3B). To investigate tumor-killing efficacy after NIR-PIT, bioluminescence imaging (BLI) was performed before and after NIR-PIT up to day seven (Figure 3E). In most mice luciferase activity decreased immediately after NIR-PIT and then gradually increased over the ensuing days (Figure 3E). This implies a large amount of initial cancer cell killing, followed by slower outgrowth of cells not originally killed by NIR-PIT. The effect of the combination group was comparable with that of CD44-targerted NIR-PIT in the BLI images. In all the treated groups, BLI after treatment was significantly lower at all time points after the initial light exposure than in the control group (*p* < 0.05, Dunnett’s test) (Figure 3F). However, there were no significant differences in luciferase activity between CTLA4 mAb alone and combination group and between CD44-targeted NIR-PIT alone and combination groups. In acute phase after treatment, there was no evidence of the success of the combination therapy compared with the single regimens. Tumor volumes in all the treated groups were significantly smaller 2, 5, 7, and 10 days after NIR-PIT compared with the control group (*p* < 0.05, Dunnett’s test) (Figure 3G). Combination group showed significantly smaller tumor volume than CD44-targeted NIR-PIT group at 12 and 17 days after NIR-PIT (*p* < 0.05, Tukey–Kramer test) (Figure 3G). In addition, combination group showed significantly smaller tumor volume than CTLA4 mAb group at 19 days after NIR-PIT (*p* < 0.05, Mann–Whitney U test) (Figure 3G). These data demonstrated that CD44-targeted NIR-PIT combined with CTLA4 blockade led to the slowest rate of tumor regrowth compared with the other treated groups. To analyze survival in long-term follow-up, Gehan–Breslow–Wilcoxon test, which assigns greater weight to early deaths, was used because CTLA4 mAb was administered only within one week after CD44-targeted NIR-PIT and early deaths played a more important role in capturing the difference in such survival characteristics between the treatment groups than late deaths in order to focus on activation of trigger on anti-tumor immunity in early phase after treatment. The combined therapy also was associated with significantly prolonged survival compared with all the other three groups (*p* < 0.05, Gehan–Breslow–Wilcoxon test) (Figure 3J). Two of 18 mice in the CTLA4 mAb group and three of 18 mice in the combination group achieved complete remission. Our results showed that CD44-targeted NIR-PIT combined with CTLA4 mAb checkpoint blockade leads to marginally improved outcome compared with the other two monotherapies for MC38-luc tumors.

### 2.4. Efficacy of CD44-Targeted NIR-PIT Combined with CTLA4 Blockade for LL/2 Tumors

The NIR-PIT regimen and imaging protocol are depicted in Figure 3A. In the NIR-PIT treated groups, anti-CD44-mAb-IR700 was administrated one day before the first light exposure (day −1), and the NIR-light was exposed over the following two days; 50 J/cm^2^ on day 0 and 100 J/cm^2^ on day one. IR700 tumor fluorescence signal decreased due to dispersion of fluorophore from dying cells and partial photo-bleaching (Figure 3C). Tumor volumes in all the treated groups were significantly smaller 2, 5, 7, and 10 days after NIR-PIT compared to that in the control group (*p* < 0.05, Dunnett’s test) (Figure 3H). Both CD44-targeted NIR-PIT and combination groups showed significantly smaller tumor volume than CTLA4 mAb group at 2, 5, 7, 10, 12, and 14 days after NIR-PIT (*p* < 0.05, Tukey–Kramer test) (Figure 3H). The CD44-targeted NIR-PIT group and combination group showed significantly greater tumor reduction compared to the CTLA4 mAb group (Figure 3H). In the long-term follow-up, the combination group had significantly prolonged survival after NIR-PIT compared with the CTLA4 mAb group (*p* < 0.05, Gehan–Breslow–Wilcoxon test) (Figure 3K), but there was no significant difference between the combination group and CD44-targeted NIR-PIT group. In LL/2 tumors, CTLA4 mAb administration did not improve in vivo therapeutic benefits over CD44-targeted NIR-PIT.

### 2.5. Efficacy of CD44-Targeted NIR-PIT Combined with CTLA4 Blockade for MOC1 Tumors

**Figure 4:**
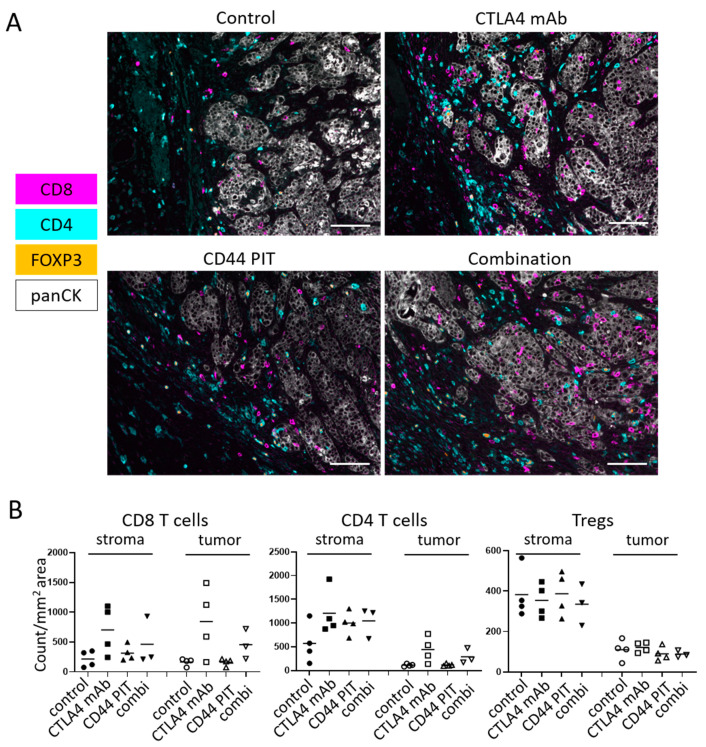
Immune cell infiltration in MOC1 tumors 1 week after NIR-PIT. (**A**) Multiplex IHC images from MOC1 tumors 7 days after treatment. Control, no treatment; CTLA4 mAb, CTLA4 immune-checkpoint blockade only; CD44 PIT, CD44-targeted NIR-PIT only; combination, CD44-targeted NIR-PIT combined with CTLA4 immune-checkpoint blockade. Expressions of CD8, CD4 and FOXP3 are shown in magenta, cyan and orange respectively. The tumor tissues were stained with anti-pan-Cytokeratin, shown in white. The representative pictures from four experiments are shown. (×200, scale bar = 100 µm). (**B**) T cell count in the tumor microenvironment. Cell number of CD8 T cells, non-regulatory CD4 T cells and Tregs in stroma and tumor tissue were counted in multiplex IHC images. Data were shown as cell count per megapixel area. Control, no treatment; CTLA4 mAb, CTLA4 immune-checkpoint blockade only; CD44 PIT, CD44-targeted NIR-PIT only; combi, CD44-targeted NIR-PIT combined with CTLA4 immune-checkpoint blockade (n = 4 for control, CTLA4 mAb and CD44 PIT, n = 3 for combi, no statistically significant difference (One-way ANOVA).

The NIR-PIT regimen and imaging protocol are depicted in Figure 3A. In the NIR-PIT treated groups, anti-CD44-mAb-IR700 was administrated one day before the first light exposure (day −1), and the NIR-light was exposed over the following two days; 50 J/cm^2^ on day 0 and 100 J/cm^2^ on day one. IR700 tumor fluorescence signal decreased due to dispersion of fluorophore from dying cells and partial photo-bleaching. (Figure 3D). Tumor volumes in all the treated groups were significantly smaller 7, 11, 14, 17, 21, 24 and 28 days after NIR-PIT compared to the control group (*p* < 0.05, Dunnett’s test) (Figure 3I). CTLA4 mAb group showed significantly greater tumor volume than combination group at 17, 21, 24 and 28 days after NIR-PIT (*p* < 0.05, Tukey–Kramer test) (Figure 3I). Among CTLA4 mAb, CD44-targeted NIR-PIT, and combination groups, combination group showed significantly smaller tumor volume than CD44-targeted NIR-PIT group at 35, 38 and 42 days after NIR-PIT, and showed significantly smaller tumor volume than CTLA4 mAb group at 42 days after NIR-PIT (*p* < 0.05, Tukey–Kramer test) (Figure 3I). In long-term follow-up, the combination therapy showed significantly prolonged survival compared to CTLA4 mAb and CD44-targeted NIR-PIT (*p*< 0.05, Gehan–Breslow–Wilcoxon test) (Figure 3L). Five of 18 mice in the CTLA4 mAb group and eight of 18 mice in the combination group achieved complete remission. Thus, combined therapy was superior to the other groups in MOC1 tumors. The histological analysis of MOC1 tumor one week after treatment showed that infiltration of CD8^+^ and CD4^+^ T-cells into the tumor bed dramatically increased after CTLA4 immune-checkpoint blockade alone and combined regimen of CD44-targeted NIR-PIT with CTLA4 immune-checkpoint blockade (Figure 4 and Supplementary Figure S1). CD44-targeted NIR-PIT also eliminates pre-existing activated immune cells in tumor beds because CD44 is expressed in not only cancer stem cells but also effector immune cells. In this study, since multiplex immunohistochemistry was technically difficult immediately after CD44-targeted NIR-PIT due to severe damage on tumor tissue that lead to ill-defined tumor boarder, we performed ex vivo CD44-targeted NIR-PIT on behalf. Ex vivo CD44-targeted NIR-PIT remarkably reduced CD44^+^ CD8 T cells isolated form tumor tissues (Supplementary Figure S2). Nonetheless, CD44^+^ CD8 T cells were found in MOC1 tumor tissues one week after combination of CD44-targeted NIR-PIT and CTLA4 mAb (Supplementary Figure S2), which suggested that newly induced T cells were accommodated and operated anti-tumor immune activity to suppress tumor growth for MOC1 tumors.

## 3. Discussion

Immune checkpoint inhibitors such as PD-1/PD-L1 and CTLA4 blockade are emerging cancer immunotherapies which block the immunosuppressive mechanisms which help tumor evade immune surveillance. Immune responses induced by checkpoint inhibition can be durable, yet only a small subset of cancer patients show clinically meaningful responses to checkpoint therapies [18]. Therefore, new cancer therapy regimens that combine checkpoint inhibition with other therapies are under investigation. NIR-PIT can potentiate the effects of checkpoint inhibition because NIR-PIT releases various cancer antigens, activates the dendritic cell system, [6], thus, inducing multi-clonal cancer antigen-specific T-cell immunity [10]. These effects are upstream of the targets of checkpoint inhibition in anti-cancer host immunity and therefore, potentially additive. We have recently reported that NIR-PIT combined with PD-1 blockade showed highly enhanced anti-cancer immunity in several syngeneic cancer models [10]. Although immune checkpoint blockade of CTLA4 axis is upper stream than the effects of PD-1 axis, NIR-PIT effects are still upstream of the targets of CTLA4 checkpoint inhibition in anti-cancer host immunity [1,3]. Therefore, newly proliferated multi-clonal cancer antigen-specific T-cells that are induced by NIR-PIT can be further activated with CTLA4 checkpoint inhibition [1,10].

Previously, we showed that MC38-luc bearing mice treated with CD44-targeted NIR-PIT and PD-1 blockade showed significantly prolonged survival and an increase in complete remission rate by the combination of CD44-targeted NIR-PIT and PD-1 blockade (70%) was extremely greater compared to PD-1 mAb (20%) or CD44-targeted NIR-PIT alone (0%) [10]. On the other hand, in this study, combination of CD44-targeted NIR-PIT and CTLA4 blockade for MC38-luc bearing mice showed significantly prolonged survival compared to CTLA4 mAb or CD44-targeted NIR-PIT alone, but an increase in complete remission rate by the combination therapy (approximately 17%) has been limited. Here we show that CD44-targeted NIR-PIT combined with CTLA4 blockade produces a lower complete remission rate than CD44-targeted NIR-PIT combined with PD-1 blockade which was reported in our previous research [10]. In LL/2 bearing mice in which CTLA4 blockade showed almost no effects, CD44-targeted NIR-PIT combined with CTLA4 blockade did not show significantly prolonged survival compared with CD44-targeted NIR-PIT alone. In the MC38 and LL/2 models which have similar CD44 expression [10], the PD-1/PD-L1 pathway is suggested to be more critical as the complementary immunosuppressive mechanism than is the CTLA4 axis. However, to clarify this, a triple combination therapy with CD44-targeted NIR-PIT, anti-PD-1 and anti-CTLA-4 might contribute to bring out some novelty.

In contrast, despite the low expression level of CD44, in MOC1 tumors CD44-targeted NIR-PIT combined with CTLA4 blockade showed significantly prolonged survival and increased rate of complete remission (44%) compared with CTLA4 mAb or CD44-targeted NIR-PIT alone. In our previous study, in MOC1 cells CD44-targeted NIR-PIT combined with PD-1 blockade did not show significantly prolonged survival compared to PD-1 blockade alone and showed complete response in only one of 13 treated mice (8%) [10]. NIR-PIT targeting CD44 selectively induces necrotic/immunogenic cell death [6], unlike the apoptotic cell death induced by most other cancer therapies [19]. Cancer cells dying by immunogenic cell death release damage associated signals including adenosine triphosphate (ATP), calreticulin and high mobility group box 1 (HMGB1) that promote maturation of immature dendritic cells (DCs) adjacent to the dying cancer cells. Cell membrane rupture induced by NIR-PIT releases fresh tumor-specific antigens in their undamaged forms into the tumor microenvironment (TME). Antigen-presenting now-mature DCs process and present multiple cancer specific antigens to naive T-cells for priming [6,10,20]. However, in some syngeneic mouse models, Tregs suppress this host anti-tumor immunity mediated by inhibiting DC function through the CTLA4 axis [21,22]. In this study, both CTLA4 immune blockage alone and combination with CD44-targeted PIT increased the T cell infiltration into MOC1 tumor seven days after the treatments, suggesting that primary immune activation was augmented by CTLA4 immune blockade. CD44 is also expressed on activated immune cells in tumor beds as well as cancer stem cells or immunosuppressive cells. Therefore, most of activated immune cells in tumor beds are eliminated immediately after CD44-targeted NIR-PIT. The combination therapy of CD44-targeted NIR-PIT and CTLA4 blockade showed only similar level of T cell infiltration in tumor beds compared with CTLA4 blockade alone one week after therapy. However, all restored T cells infiltrated in tumor beds after the combination therapy are newly-accommodated cells. Among them, large number of CD44^+^ CD8 T cells were observed in MOC1 tumors. The improved long-term survival suggests that the combination therapy successfully established long term immunity against MOC1 tumor. In MOC1 tumors, CD44-targeted NIR-PIT might have destroyed smaller number of tumor cells to trigger immune reaction, but combination with CTLA4 blockade might overcome immunosuppression and enhanced anti-tumor responses against MOC1 cells enough to achieve complete remissions along with smaller tumor volume after the combination therapy than CTLA4 blockade alone. In this study, MOC1 tumor before therapy showed relatively low density of Tregs in TME compared with MC38-luc and LL/2 tumors. Tumor types with low Treg density might associate with effective therapeutic efficacy mediated by CTLA4 blockade, but further studies such as comparison of pre- and post-treatment Treg densities in different kinds of tumor types are required to elucidate this hypothesis. Alternatively, it is known that myeloid derived suppressor cells (MDSCs), found in MOC1 tumors can inhibit immune response [23,24]. In particular, granulocytic MDSCs (gMDSCs) accumulate with tumor progression and correlate with depletion of effector immune cells [25]. MOC1 tumors show higher cellular distribution of gMDSCs compared with MC38-luc and LL/2 tumors [26,27]. Administration of CTLA4 mAb blocks the immunosuppressive mechanism of gMDSCs as well as Treg cells within the tumor microenvironment in MOC1 tumors [25], which may further confer therapeutic benefits. There is variation of T cell distribution among different tumor types (Figure 2B). Taken together, these data confirm that the individual TME of each tumor varies and therefore, the most effective immune checkpoint targets to use in combination with NIR-PIT also varies with tumor type based on cell line because tumor tissues in different cell lines are composed of a variety of immune cell components [26]. However, further study is required to elucidate the mechanism of NIR-PIT combined with immune checkpoint blockade, including comparison of immune cell infiltration in pre- and post-treatment status for different types tumors.

This study has several limitations. First, we used subcutaneously ectopic tumor models. Although many tumor immunology studies use this model, an orthotopic model might be considered more clinically relevant [28]. However, in the present study, it was important that a consistent size, shape, and location of each tumor be maintained to enable a fair comparison of tumor growth. The orthotopic model can produce more variable results depending on how well the tumor is implanted within the organ. Thus, we opted for a simpler and more reliable subcutaneous tumor model. Second, CD44 is also expressed on activated immune cells within the tumor treatment field, raising the concern that CD44-targeted NIR-PIT could also eliminate some proportion of desirable effector immune cells. NIR-PIT with an antibody with high specificity for mouse tumors is therefore more favorable to use in syngeneic mouse models of cancer. A few such antibodies including anti-EGFR are commercially available, yet very expensive for planning therapeutic experiments.

## 4. Materials and Methods

### 4.1. Cell Culture

MC38 cells (murine colon cancer), which were generously provided by Dr. Thomas Waldmann, NIH [29] stably expressing luciferase (MC38-luc, generated via stable transduction with RediFect Red-Fluc lentivirus from PerkinElmer per manufacturer recommendations), LL/2 cells (murine Lewis lung carcinoma), which was purchased from ATCC (Rockville, MD, USA), and MOC1 cells (murine oral carcinoma), which were produced and generously provided by Dr. Clint Allen, NIH [30] were used in this study. High luciferase expression on the MC38-luc cells was confirmed through 10 passages. MC38-luc and LL/2 cells were cultured in RPMI1640 supplemented with 10% FBS and 1% penicillin–streptomycin (all Gibco brand, ThermoFisher Scientific, Waltham, MA, USA) in tissue culture flasks (182 cm^2^; CELLTREAT Scientific Products, Pepperell, MA, USA) in a humidified incubator at 37 °C in an atmosphere of 95% air and 5% carbon dioxide. MOC1 cells were cultured in HyClone Iscove’s modified Dulbecco’s medium (GE Healthcare Life Sciences, Marlborough, MA, USA)/HyClone Ham’s Nutrient Mixture F12 (GE Healthcare Life Sciences, Marlborough, MA, USA) at a 2:1 mixture with 5% FBS, 1% penicillin/streptomycin, 3.5 ng/mL EGF (EMD Millipore Corporation, Burlington, MA, USA), 40 ng/mL hydrocortisone (Sigma-Aldrich, St. Louis, MO, USA), and 5 ng/mL insulin (Sigma-Aldrich, St. Louis, MO, USA) in the tissue culture flasks in a humidified incubator at 37 °C in an atmosphere of 95% air and 5% carbon dioxide. Cells were authenticated via in vitro growth characteristics.

### 4.2. Reagents

Water soluble, silica-phthalocyanine derivative, IRDye700DX NHS ester was obtained from LI-COR Bioscience (Lincoln, NE, USA). An anti-mouse/human CD44 mAb (IM7) and an anti-mouse CTLA4 mAb (9D9, mouse IgG2b) were purchased from BioXCell.

### 4.3. Synthesis of IR700-Conjugated Anti-CD44 mAb

Anti-CD44-mAb (1mg, 6.7 nmol/L) was incubated with IR700 (65.1 μg, 33.3 nmol, 10 mmol/L in DMSO) and 0.1 mol/L Na_2_HPO_4_ (pH 8.5) at room temperature for 1 h. The mixture was purified with a gel filtration column (Sephadex G 25 column, PD-10, GE Healthcare, Piscataway, NJ, USA). The protein concentration was determined with Coomassie Plus protein assay kit (Thermo Fisher Scientific Inc, Rockford, IL, USA) by measurement of the absorption at 595 nm with spectroscopy (8453 Value System; Agilent Technologies, Santa Clara, CA, USA). We abbreviate IR700-conjugated anti-CD44 mAb as anti-CD44-mAb-IR700.

### 4.4. In Vitro NIR-PIT

MC38-luc, LL/2, or MOC1 cells (2 × 10^5^) were seeded into 12-well plates, incubated for 24 h, and then exposed to media containing anto-CD44-mAb (10 mg/mL) for 6 h at 37 °C. Cells were irradiated with a red light-emitting diode (LED, 690 ± 20 nm wavelength, L690-66-60; Marubeni America Co., Houston, TX, USA) at a power density of 50 mW/cm^2^. Cells were scratched after treatment, stained with propidium iodide (PI, 2 mg/mL) at room temperature for 30 min, and then assessed for PI positivity on a BD FACS Calibur (BD Biosciences, San Jose, CA, USA) using CellQuest software.

### 4.5. Animal Model

All procedures were performed in compliance with the Guide for the Care and Use of Laboratory Animals and approved by the National Cancer Institute Animal Care and Use Committee (MIP-003). Six- to eight-week-old female C57BL/6 mice (strain #000664) were purchased from the Jackson laboratory. The lower part of the body of the mice was shaved for irradiation and image analysis. Mice with tumors reaching approximately 150 mm^3^ in volume were used for the experiments. Tumor volumes were calculated from the greatest longitudinal diameter (length) and the greatest transverse diameter (width) using the following formula; tumor volume = length × width^2^ × 0.5, based on caliper measurements. Mice were monitored each day and tumor volumes were measured three times a week for MC38-luc and LL/2 tumors and twice a week for MOC1 tumors until the tumor volume reached 2000 mm^3^, whereupon the mice were euthanized with inhalation of carbon dioxide gas. Tumor disappearance for 4 weeks or longer after treatment was defined as complete remission.

### 4.6. In Vivo NIR-PIT

MC38-luc cells (8 million), LL/2 cells (8 million) and MOC1 cells (4 million) were subcutaneously injected in the dorsum of mice. When the tumors reached volumes of approximately 150 mm^3^ they were divided randomly into 4 experimental groups for the following treatments: (1) no treatment (control); (2) intravenous injection of 100 μg anti-CD44-mAb-IR700 (day −1) followed by intraperitoneal injection of 100 μg anti-CTLA4-mAb on day −1, 1, 3 and 5 without exposure to NIR light (CTLA4 mAb); (3) intravenous injection of 100 μg anti-CD44-mAb-IR700 (day −1) followed by external NIR light irradiation at 50 J/cm^2^ on day 0 and 100 J/cm^2^ on day 1 (24 and 48 h after anti-CD44-mAb-IR700 injection, respectively) (CD44-targeted NIR-PIT); and (4) intravenous injection of 100 μg anti-CD44-mAb-IR700 (day −1) followed by external NIR light irradiation at 50 J/cm^2^ on day 0 and 100 J/cm^2^ on day 1 (24 and 48 h after anti-CD44-mAb-IR700 injection, respectively) with intraperitoneal injection of 100 μg anti-CTLA4-mAb on day −1, 1, 3 and 5 (combination). For the mice with MC38-luc tumor, LL/2 tumor, and MOC1 tumor in the NIR-PIT treated groups, intravenous injection of the APCs was performed 7, 7, and 28 days after tumor inoculation, respectively. NIR light was administered to tumor-bearing mice using a red light emitting diode (LED), which emits light in the range of 670 to 710 nm wavelength (L690-66-60; Marubeni America Co., Houston, TX, USA) at a power density of 50 mW/cm^2^ as measured with an optical power meter (PM 100, Thorlabs, Newton, NJ, USA). IR700 absorbs light at approximately 690nm. IR700 fluorescence images were obtained before and after therapy.

### 4.7. In Vivo BLI and IR700 Fluorescence Imaging

To obtain BLI images in MC38-luc tumor–bearing mice, D-luciferin (15 mg/mL, 150 mL; Goldbio, St. Louis, MO, USA) was intraperitoneally injected into mice. Luciferase activity was analyzed with a BLI system (Photon Imager; Biospace Lab, Nesles-la-Vallée, France) using relative light units. Regions of interests (ROIs) were placed over the entire tumor. The counts per minute of relative light units were calculated using M3 Vision Software (Biospace Lab, Nesles-la-Vallée, France) and converted to the percentage decrease in light based on a comparison of post NIR-PIT to pre-therapy BLI using the following formula: (relative light units after treatment)/(relative light units before treatment) × 100 (%); [31]. BLI was performed before and after NIR-PIT (protocol above) on day 0 to day 7. In vivo IR700 fluorescence images were obtained with a Pearl Imager (LI-COR Biosciences, Bad Homburg vor der Höhe, Germany) with a 700-nm fluorescence channel.

### 4.8. Multiplex Immunohistochemistry (IHC)

The multiplex immunohistochemistry (IHC) staining was performed using BOND RXm automated stainer (Leica Biosystems, Buffalo Grove, IL, USA). The paraffin-embedded tumor samples were sliced into 4-μm sections. Dewaxed sections were incubated in BOND ER2 solution (EDTA based, pH 9.0; Akoya Bioscience, Marlborough, MA, USA) at 95 °C for 20 min for antigen retrieval treatment, followed by multiplex antibody staining using the Opal 7-Color Automation IHC Kit (Akoya Bioscience, Marlborough, MA, USA). Following antibodies were used; anti-CD44 (clone IM7; Bio X Cell; 1:1000 dilution), anti-CD8 (clone EPR20305; Abcam 1:500 dilution), anti-CD4 (clone EPR19514; Abcam 1:1000 dilution), anti-FOXP3 (clone 1054C; Novus Biologicals 1:1000 dilution) and anti-pan-Cytokeratin (rabbit polyclonal; Bioss 1:500 dilution). For secondary antibody, ImmPRESS HRP Polymer Detection Kit of either anti-rat IgG or anti-rabbit IgG format (Vector Laboratories, Peterborough, UK) were used. Stained slides were mounted with Vectashield antifade mounting media (Vector laboratories, Peterborough, UK) then images were obtained using Mantra Quantitative Pathology Workstation (Akoya Bioscience, Marlborough, MA, USA). The multiplex IHC images were analyzed using inForm Tissue Finder software (Akoya Biosystems, Marlborough, MA, USA). Cell phenotypes were defined based on the antigen expressions as followings; membrane CD8^+^ = CD8 T cell, membrane CD4^+^/nuclear FOXP3^−^ = non-regulatory CD4 T cells, membrane CD4^+^/nuclear FOXP3^+^ = regulatory T cell (Treg). Tissue phenotypes were determined as followings; pan-Cytokeratin positive = tumor, other tissue around the tumor = stroma. At least five pictures were taken for each tumor sample, both cell phenotype count and tissue phenotype area were added up for all pictures from the same tumor to calculate cell count per area.

### 4.9. Flow Cytometry

In vitro, MC38-luc, LL/2, or MOC1 cells (2 × 10^5^) were incubated with anti-CD44-mAb-IR700 (10 µg/mL) for 15 min at 4 °C. To validate specific binding, unconjugated anti-CD44 of 10-fold molar excess was added to some samples 15 min prior to the incubation with anti-CD44-mAb-IR700. Fluorescence of the cells were analyzed with FACSCalibur and FlowJo software. Dead cells were excluded from the analysis based on the staining with LIVE/DEAD Stain Kit (Thermo Fisher Scientific, Waltham, MA, USA).

For the ex vivo NIR-PIT, tumor, lymph node and spleen were isolated from MC38-luc tumor bearing mouse and single cell suspension were prepared as followings; tumor was digested with collagenase type IV (1mg/mL, Thermo Fisher Scientific Waltham, MA, USA) and DNase I (20 μg/mL, Millipore Sigma, Burlington, MA, USA) in 37 °C for 20 min then physically dissociated on the 70 μm cell strainer. Lymph node and spleen were gently mashed on the petri dish and were passed through 70 μm cell strainer. Single cell suspensions were aliquoted into 24 well cell culture plate and incubated in RPMI + 10% FBS containing 2 μg/mL anti-CD44-mAb-IR700 for 1 h then 5 J/cm^2^ of NIR light was irradiated using LED light source. After 1 h incubation, cells were stained with following antibodies; anti-CD44 (clone KM201, Thermo Fisher Scientific, Waltham, MA, USA), anti-CD3e (clone 145-2C11, eBioscience, San Diego, CA, USA) and anti-CD8 (clone 53-6.7, Thermo Fisher Scientific, Waltham, MA, USA). Dead cells were stained with LIVE/DEAD™ Fixable Far Red Dead Cell Stain (Thermo Fisher Scientific, Waltham, MA, USA). The stained cells were analyzed with FACSCalibur (BD) and the data were analyzed with FlowJo software (BD).

### 4.10. Statistical Analysis

Quantitative data were expressed as means ± SEM. The Mann–Whitney U test was used to compare differences between two groups. For multiple comparisons (≥3 groups), comparison to a known control group was analyzed with Dunnett’s test and in other cases, a one-way analysis of variance followed by the Tukey–Kramer test was used. The cumulative probability of survival was analyzed with Kaplan–Meier survival curves and the results were compared using the Gehan–Breslow–Wilcoxon test. Statistical analysis was performed with JMP 13 software (SAS Institute, Cary, NC, USA). A *p* value of less than 0.05 was considered significant.

## 5. Conclusions

CD44-targeted NIR-PIT combined with CTLA4 blockade demonstrates superior in vivo therapeutic efficacy to either CD44-targeted NIR-PIT or CTLA4 mAb alone in MC38-luc and MOC1 tumors. CD44-targeted NIR-PIT combined with CTLA4 blockade showed more effective tumor killing and complete remission in MOC1 tumors than NIR-PIT combined with PD-1 blockade in a previous study whereas in MC38-luc and LL/2 tumors NIR-PIT combined with PD-1 blockade was more effective. Selection of the most appropriate checkpoint inhibitor to combine with NIR-PIT may vary among different syngeneic tumor models and is therefore, likely to vary in human tumors as well.

## Supplementary Materials

The following are available online at https://www.mdpi.com/2076-393X/8/3/528/s1, Figure S1: Separate channel images from the pictures in Figure 4, Figure S2: CD44 expression in CD8 T cells before and after treatment.
