# Supplementary material for: Near-Infrared Photoimmunotherapy Combined with CTLA4 Checkpoint Blockade in Syngeneic Mouse Cancer Models
Source: Vaccines (Basel). 2020 Sep 14;8(3):528. doi: 10.3390/vaccines8030528 (PMC7564971; doi:10.3390/vaccines8030528)
Supplement: Supplementary file 1 [file vaccines-08-00528-s001.pdf]

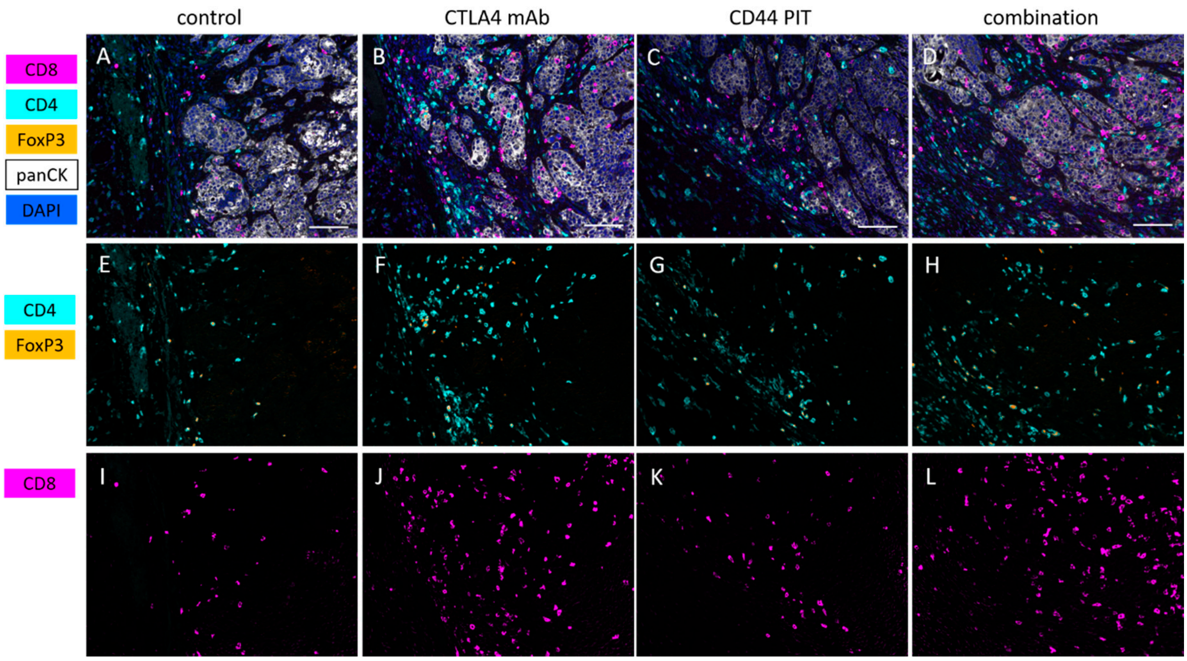

Supplemental Figure S1. Separate channel images from the pictures in Figure 4. A-D. composite images of CD8, CD4, FOXP3, pan-Cytokeratin and DAPI staining. E-H. composite images of CD4 and FOXP3 staining. I-L. Single channel images of CD8 staining.

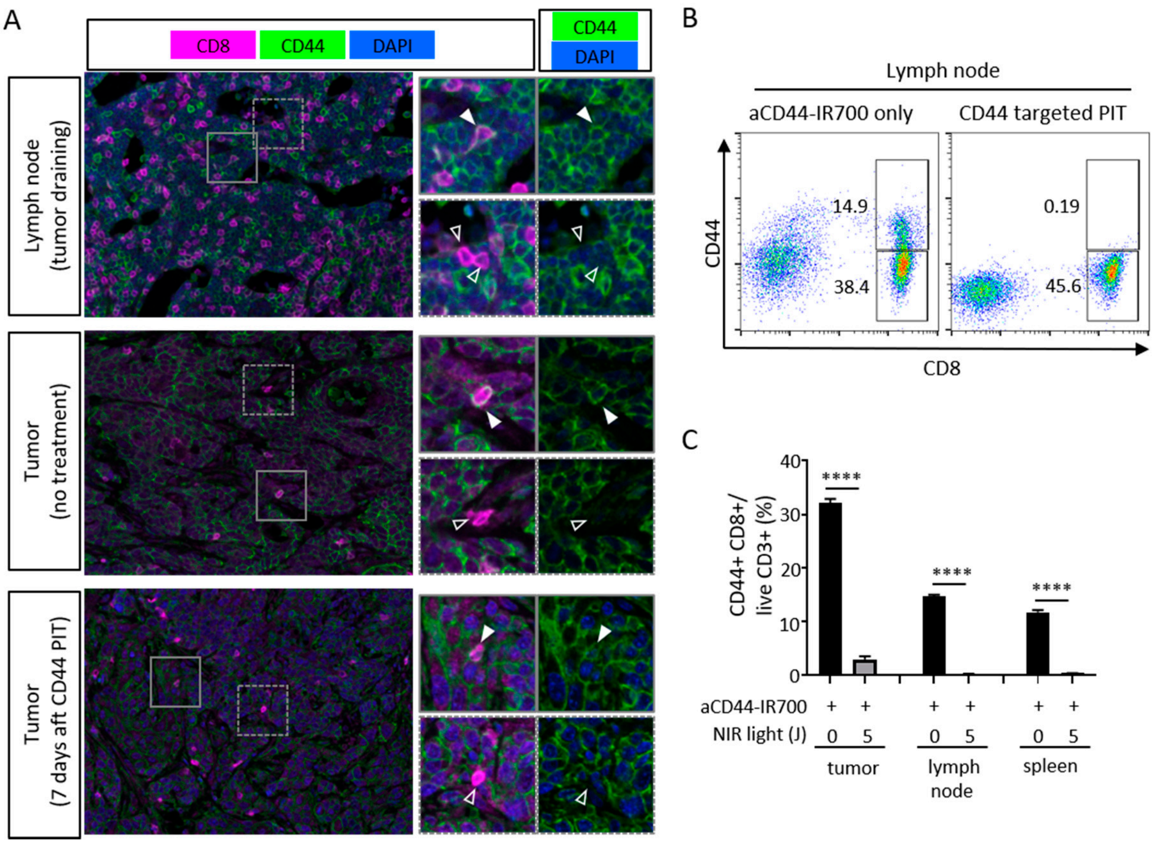

Supplemental Figure S2. CD44 expression in CD8 T cells before and after treatment. A. CD44 expression in CD8 T cells was analyzed with multiplex IHC in tumor draining lymph nodes, MOC1

tumors with no treatment and tumors 7 days after CD44-targeted NIR-PIT. CD8 and CD44 expression are shown in magenta and green respectively. DAPI is shown in blue. Examples of $CD44^+$ $CD8^+$ T cells and $CD44^-$ $CD8^+$ T cells are shown in squares in solid line and broken line respectively. Enlarged pictures are shown in the right panels. Examples of CD44+ $CD8^+$ T cells and $CD44^-$ $CD8^+$ T cells are indicated with filled arrowhead and open arrowhead respectively. B, C. FACS analysis after ex-vivo CD44-targeted NIR-PIT. A tumor, a draining lymph node and a spleen are collected from a MC38-luc tumor bearing mouse and subjected to CD44-targeted NIR-PIT. B. Representative dot plot of CD44 and CD8 expression in live/$CD3^+$ gate of lymph node samples. Note that $CD44^+$/$CD8^+$ population disappeared after CD44-targeted NIR-PIT. C. Percentages of $CD44^+CD8^+$ T cells in live $CD3^+$ gate shown as mean ± SEM (n = 5; ****, $p < 0.001$; vs. NIR light 0J for each tissue).
